# Electric field strength induced by electroconvulsive therapy is associated with clinical outcome

**DOI:** 10.1016/j.nicl.2021.102581

**Published:** 2021-02-09

**Authors:** Egill Axfjord Fridgeirsson, Zhi-De Deng, Damiaan Denys, Jeroen A. van Waarde, Guido A. van Wingen

**Affiliations:** a Department of Psychiatry, Amsterdam UMC, University of Amsterdam, Amsterdam Neuroscience, Amsterdam, the Netherlands; b Amsterdam Brain and Cognition, University of Amsterdam, Amsterdam, the Netherlands; c Noninvasive Neuromodulation Unit, Experimental Therapeutics & Pathophysiology Branch, National Institute of Mental Health, United States; d Department of Psychiatry, Rijnstate Hospital, Arnhem, the Netherlands; e The Netherlands Institute for Neuroscience, an Institute of the Royal Netherlands Academy of Arts and Sciences, Amsterdam, the Netherlands

**Keywords:** Electroconvulsive therapy, Major depressive disorder, Finite element modelling

## Abstract

The clinical effect of electroconvulsive therapy (ECT) is mediated by eliciting a generalized seizure, which is achieved by applying electrical current to the head via scalp electrodes. The anatomy of the head influences the distribution of current flow in each brain region. Here, we investigated whether individual differences in simulated local electrical field strength are associated with ECT efficacy. We modeled the electric field of 67 depressed patients receiving ECT. Patient’s T1 magnetic resonance images were segmented, conductivities were assigned to each tissue and the finite element method was used to solve for the electric field induced by the electrodes. We investigated the correlation between modelled electric field and ECT outcome using voxel-wise general linear models. The difference between bilateral (BL) and right unilateral (RUL) electrode placement was striking. Even within electrode configuration, there was substantial variability between patients. For the modeled BL placement, stronger electric field strengths appeared in the left hemisphere and part of the right temporal lobe. Importantly, a stronger electric field in the temporal lobes was associated with less optimal ECT response in patients treated with BL-ECT. No significant differences in electric field distributions were found between responders and non-responders to RUL-ECT. These results suggest that overstimulation of the temporal lobes during BL stimulation has negative consequences on treatment outcome. If replicated, individualized pre-ECT computer-modelled electric field distributions may inform the development of patient-specific ECT protocols.

## Highlights

• Finite element modelling is used to estimate the electric field induced by ECT.
• The electric field induced by ECT is highly variable between patients.
• The electric field in certain brain regions is associated with less optimal ECT-outcome.
• White matter distribution in temporal lobes seems important for this relationship.

## 1 Introduction

Electroconvulsive therapy (ECT), a form of transcranial electrical stimulation (TES), is a highly effective treatment, especially in patients suffering major depressive episodes (MDE). The majority of MDEs occur in patients with major depressive disorder (MDD); in bipolar disorder (BD) the number of lifetime MDEs is higher (Moreno et al., 2012). MDEs have a lifetime prevalence of 15% in high income countries (Kessler and Bromet, 2013), and the World Health Organization ranks MDD as one of the leading causes of disability (Ferrari et al., 2013, Ustün et al., 2004) with BD not far behind (Ferrari et al., 2016). When using ECT in patients with MDEs, remission rates are reached ranging from 48% for patients with previous pharmacotherapy failure up to 65% for patients without pharmacotherapy-resistance (Heijnen et al., 2010, Lisanby, 2007, Prudic et al., 2004). However, patients undergoing ECT may suffer from cognitive side-effects, most often post-ictal confusion directly after the ECT-session, anterograde amnesia (the patient does not ‘save’ memories) and sometimes (severe) retrograde amnesia (the patient actually ‘forgets’ memories) (Geddes et al., 2003).

All treatment modalities with TES (e.g., ECT, transcranial direct current stimulation [tDCS] and transcranial alternate current stimulation [tACS] (Saturnino et al., 2019) induce electric fields in the human brain, but in ECT much higher electrical currents are used - reaching larger brain areas - compared to tDCS and tACS. During each ECT-session, under general anesthesia with muscle relaxant, a train of electrical pulses is applied to the scalp of the patient to induce tonic-clonic seizures. Between two electrodes, an in advance estimated total charge is administered bidirectionally. Modern-day ECT uses constant current (0.8 or 0.9 Ampere) administered in brief pulses (typically with a pulse width of 0.25–1.5 ms) in varying frequencies and stimulus train durations (typically up to 8 s) (Peterchev et al., 2010). In order to be clinically effective, the induced seizure activity should be elicited using stimuli substantially exceeding an individual seizure threshold (Sackeim et al., 1993). In addition to the stimulus parameters, the choice of the electrode placement is also a crucial determinant of effectiveness and cognitive side-effects. In clinical practice, several positions are in use, such as the right unilateral (RUL), bifrontal, and bilateral (BL) electrode placements, delivering the therapeutic electrical charge differently to the patients’ brain.

During an ECT-stimulus, electrical current flows throughout the scalp, skull, cerebrospinal fluid, and brain. In vivo measurements of induced electric field strengths in humans and non-human primates have shown that the maximum electric fields in humans reach up to 0.5 V/m for 1 mA stimulation currents (Opitz et al., 2016), which is in the range predicted by realistic head model simulations. Another study reported maximum cortical electric field strengths of 0.4 V/m for 1 mA (Huang et al., 2017). Huang et al. (Huang et al., 2017) also reported a good spatial correlation between the measured electric fields and simulated values based on realistic head models derived from individual MRIs. The proportion of current entering the human brain is highly individual and depends on skull thickness, unique head and brain anatomy as well as the electrical resistances of the consecutive compartments of the human head (Sackeim et al., 1994, Deng et al., 2015). After reaching brain tissue, the current traverses the gray matter relatively freely, but in the white matter it is biased to flow along the directions of the axons since the transverse resistance of white matter fibers is higher than the longitudinal resistance (Nicholson, 1965, Geddes and Baker, 1967). Also, the strength of this effect varies substantially depending on factors such as electrode placement and surrounding individual brain anatomy (ventricles, blood vessels, bony structures). Furthermore, substantial variation of seizure thresholds occurs in different human brain areas (Lesser et al., 1984) and it was suggested that brain regions associated with ECT efficacy (e.g., prefrontal areas) may be distinct from regions critical to the development of cognitive side-effects (e.g., medial temporal lobes) (Nobler and Sackeim, 2008, Abrams and Taylor, 1976, Ottosson, 1960, Sackeim, 2004). Consequently, the electric field distribution by the used electrode placement may be a determinant of beneficial generalized seizure activity in patients, as well as the risk of cognitive side-effects.

To choose the supposed optimal electrode placement for the patient, clinicians consider the need for rapid and most efficient symptom release (e.g., BL placement in case of suicidality, psychosis, catatonia and/or poor physical condition) and the option to prevent (severe) cognitive side-effects (e.g., using RUL instead of BL placement) (Sackeim et al., 2007). Moreover, a substantial amount of RUL treated patients do not recover optimally and the clinical guidelines then advise to switch to BL electrode placement for maximal effectivity (Rosa et al., 2006, van et al., 2013, Sackeim et al., 2008). Daily clinical practice of ECT is still hampered by not knowing in advance the most effective and tolerable electrode placement, electrical charge and pulse width for the individual patient.

It has been hypothesized that the distribution of electric field within the brain is both associated with efficacy and cognitive side-effects (Sackeim, 2004). Recent studies using anatomically realistic head models of the electric field distribution show that BL stimulation compared to RUL stimulation will result in higher median magnitude of electric field in the whole brain as well as in deep-midline structures and temporal and inferior frontal regions (Bai et al., 2017, Lee et al., 2012). Recently a large study in 151 patients receiving RUL ECT found that the electric field causes volumetric changes in the amygdala and hippocampus (Argyelan et al., 2019). However, they did not find an association with the clinical outcome. In this current study, we extended the sample with BL-stimulated patients, and first assessed the influence of electrode placement on modelled electric field distributions in the brain in a cohort of patients suffering severe MDE. Next, we test whether these electric field distributions were associated with ECT-outcome.

## 2 Methods

### 2.1 Patients

Structural MRI and ECT-outcome data were available from patients who participated in a prospective observational study (van et al., 2013, Van Waarde et al., 2013) at Rijnstate Hospital, Arnhem, the Netherlands. All patients suffered from a severe and/or treatment-resistant MDE, according to the Diagnostic and Statistical Manual of Mental Disorders (DSM-IV-TR) as was classified by at least two independent experienced psychiatrists. Age, sex, total administered ECT-sessions during the ECT-course, and concomitant medication use were documented. Depression severity was scored by a trained research nurse, using the Montgomery–Åsberg Depression Rating Scale (MADRS) in the week before the first ECT-session and within one week after the last ECT-session. Patients with a reduction in MADRS-score after the total ECT-course of at least 50% were considered ‘responders’. The local Medical Ethical committee approved the study protocol and after a complete description of the study to the subjects, we obtained written informed consent from all participants (Registration number: NL24697.091.09).

### 2.2 Electroconvulsive therapy

ECT was administered using a constant current (0.9 A), (ultra-)brief pulse ECT device with a maximum output of 1008 mC (Thymatron IV, Somatics Incorporation, Lake Bluff, IL, USA). Pulse width was 0.25 ms in RUL ECT and 0.5 ms in BL ECT. Anesthesia was induced intravenously with etomidate (0.2–0.5 mg kg^−1^ body mass), muscle paralysis with succinylcholine (0.5–1 mg kg^−1^ body mass) intravenously and appropriate oxygenation was used (100% oxygen, positive pressure) until resumption of spontaneous respiration. Electrode placement was started RUL, except in patients with high risk for suicide and/or somatic complications or in cases where previous BL ECT had been successful. The electrodes that were used measured 5 cm in diameter. Initial seizure threshold (IST) was measured at the first ECT-session using an empirical titration method (van et al., 2013). Subsequently, the therapeutic ECT dose was set at 2.5 times IST for BL stimulation and 6 times IST for RUL stimulation. ECT was administered twice a week. RUL placement was changed into BL placement, based on a clinical decision by experienced psychiatrists if the patient did not respond after six RUL sessions. The ECT-course was terminated or lowered in frequency if the patient had been recovered (MADRS score ≤ 10) or showed no further clinical improvement over a period of two weeks, or had shown no improvement after ten BL ECT-sessions.

### 2.3 MRI acquisition

A structural magnetic resonance image (MRI) of the head was acquired within two weeks before the first ECT-session. A 1.5 T Philips MRI scanner (Philips, Best, the Netherlands) was used using a 8-channel SENSE head coil. Scanning protocol included a 1.1 mm isotropic T1-weighted turbo field echo scan with repetition time of 7.6 ms, echo time 3.5 ms, flip angle 15° and 145 saggital slices.

### 2.4 Electric field modelling

We computed the ECT-induced electric field with SimNIBS 2.1.1 (Thielscher et al., 2015). Individual patient T1-weighted images were segmented and meshed into finite-element head models using *SimNIBS:headreco* which uses the SPM12 with the CAT12 toolbox for segmentation. A representative example of the segmentation can be seen in supplementary Fig. 1. SPM12 with the CAT12 toolbox is superior to Freesurfer or SPM12 only segmentation routines (Nielsen et al., 2018). We assigned isotropic conductivity values: white matter, 0.126 S/m; gray matter, 0.275 S/m; cerebrospinal fluid, 1.654 S/m; bone, 0.01 S/m; scalp, 0.465 S/m; eyes, 0.5 S/m. Using SimNIBS’s graphical user interface, disc electrodes of 5-cm diameter were centered according to the standard RUL and/or BL ECT electrode placements (Lisanby, 2007). We solved for the field distribution first with an electrode current of 1 mA, then scaled by 900 to match the output of the Somatics Thymatron System IV device.

**Fig. 1:**
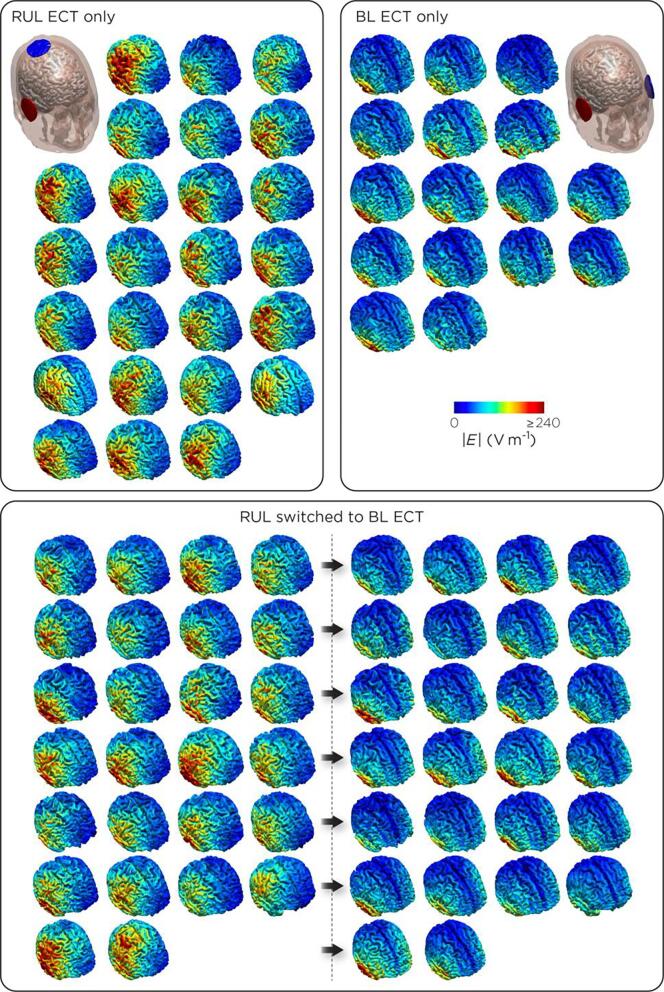
Distribution of modeled electric fields In patients treated with right unilateral (RUL) ECT only (n = 25), bilateral (BL) ECT only (n = 16), and who started using RUL electrode placement and switched to BL electrode placement (N = 26).

### 2.5 Normalization of head models

T1-weighted images were brain extracted and normalized to MNI space using advanced normalization tools (ANTs, http://stnava.github.io/ANTs/) (Avants et al., 2011, Avants et al., 2008). Then, the normalization transformation was applied to the electric field models so they were transformed to MNI space.

### 2.6 Statistics

To assess the influence of the different electrode placements, we used the data obtained from patients that had switched from RUL to BL. We subtracted the RUL electric field magnitude from the BL electric field magnitude, so the patients were their own comparison. This difference was then permuted 5000 times in FSL randomize using sign flipping to control for multiple comparisons (p < 0.05) (Winkler et al., 2014) with threshold free cluster enhancement (TFCE) (Smith and Nichols, 2009). This shows in which voxels the electric field magnitude due to one electrode configuration was significantly higher than in the other given the same stimulation current.

To explore the relationship between spatial electric field strength in ECT and outcome, a voxel-wise general linear model (GLM) was set up with the electric field strength as the independent variable and the outcome as the dependent variable. Our method, in which we used voxel-wise data as independent variable and a clinical variable as dependent variable, is the inverse of what is common for neuroimaging analysis. Also, this analysis is not implemented in any of the neuroimaging analysis software packages that are currently available. Therefore, a pseudoinverse was required to be calculated for each voxel instead of once, so a computationally efficient routine was made in the linear algebra library Eigen in C++ (https://eigen.tuxfamily.org/) to solve the least squares problem for each voxel. Then, this routine was called from a python pipeline incorporating TFCE and permutation testing. Age, sex and baseline MADRS-score were included to account for demographic variables and differences in severity at baseline. Since the electric field modelling only accounts for one pulse of one session the total number of sessions was included as a covariate. And since each patient receives a unique amount of dose (number of pulses) each session, which is a fixed multiple of the seizure threshold, the mean seizure threshold during the ECT-course was included as a covariate. For the RUL case, because no intermediate MADRS-score was available for the switchers at the time of switching from RUL to BL, we used treatment response as binary dependent variable (switchers were scored as ‘non-response’). For the BL analysis that included patients undergoing only BL stimulation as well as patients who had switched from RUL to BL stimulation, we used the end-MADRS-score as the outcome. To account for whether patients started with BL stimulation or switched from RUL stimulation, a binary covariate was included. Baseline-MADRS-scores were mean imputed for two patients for whom baseline scores were missing. We used non-parametric permutation testing with 20,000 iterations and a p-value threshold of 0.05.

## 3 Results

### 3.1 Patient characteristics and ECT-outcome

In total, 67 patients were included of which 25 were treated only with RUL electrode placement, 16 with only BL placement and 26 patients switched from RUL to BL placement. Of the 67 patients, 46 (65.6%) showed a response (MADRS ≥ 50% reduction) after the ECT-course. For the patients who were only treated with RUL, 21 (84%) patients showed response; in BL ECT only, 14 (88%) responded and 11 switchers (42.3%) responded. There was a statistically significant difference of the end-MADRS score between groups (p < 0.001). Post hoc tests showed this was driven by worse outcome in the switchers group. Additionally, the BL group had a higher initial seizure threshold (p < 0.001) and was more likely to have had previous ECT (p < 0.001). Additional patients’ characteristics are provided in Table 1.

**Table 1:** Patients’ characteristics and outcome measures.

Electrode placement	**All (n = 67)**	**Only BL^3^ (n = 16)**	**Only RUL^2^ (n = 25)**	**Switchers (n = 26)**	**P-value**
Mean age (±SD^1^), in years	58 (±15)	58 (±19)	57 (±13)	57 (±14)	0.89
Females, (%)	41 (61%)	11 (69%)	17 (68%)	13 (50%)	0.32
Baseline MADRS^4^-score (±SD)	35.7 (±8.3)	37.0 (±7.5)	36.2 (±9.7)	34.3 (±7.4)	0.56
End MADRS-score (±SD)	12.8 (±9.5)	10.3 (±9.3)	8.5 (±7.0)	18.6 (±9.1)	**<0.001**
Responders, MADRS decrease ≥ 50%, (%)	46 (66%)	14 (88%)	21 (84%)	11 (42%)	**0.001**
Remitters, MADRS at end ≤ 10 (%)	34 (51%)	9 (56%)	18 (72%)	7 (27%)	**0.005**
Initial seizure threshold in milliCoulomb (±SD)	55.3 (±34.3)	85.0 (±53.6)	47.4 (±19.7)	44.6 (±16.4)	**<0.001**
Previous ECT (%)	21 (31%)	12 (80%)	5 (20%)	4 (15.3%)	**<0.001**
Total sessions (±SD)	17.8 (±7.5)	16.6 (±5.6)	14.4 (±7.6)	21.8 (±6.7)	**<0.001**

1 Standard deviation; ^2^Right unilateral electrode placement; ^3^bilateral electrode placement; ^4^Montgomery-Åsberg Depression Rating Scale.

### 3.2 Simulated electric fields in RUL and BL ECT

In Fig. 1, the electric field distribution is shown for all patients individually. The difference between RUL and BL electrode placement was striking, and even within electrode configuration, there is substantial variability between patients.

**Fig. 2:**
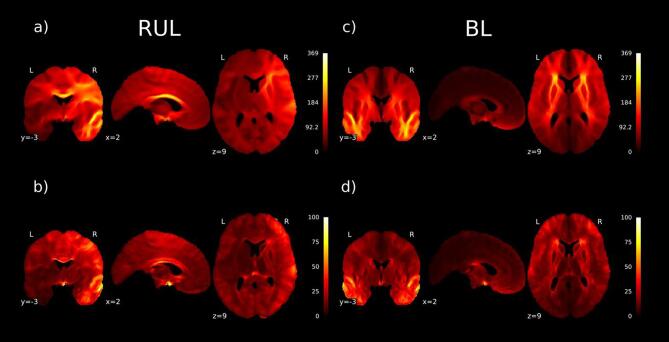
Mean and variance of electric fields RUL: The highest values and most variance of the electric fields are seen in the right hemisphere and in the corpus callosum: a) Mean magnitude of electric field in the RUL treated patients (V/m); b) Standard deviation of magnitude of electric field in RUL treated patients (V/m). BL: The highest electric field magnitude is in the white matter of both temporal lobes and white matter connecting the frontal lobes including external and internal capsules as well as the anterior corona radiate: c) Mean magnitude of electric field (V/m) for BL treated patients; d) Standard deviation of electric field in BL treated patients (V/m).

For RUL treated patients, the mean and standard deviation of the RUL electric field is shown in Fig. 2a and 2b. The highest values and most variance of the electric fields are seen in the right hemisphere and in the corpus callosum. For BL treated patients, the mean electric field and standard deviation are illustrated in Fig. 2c and 2d. As is shown, in BL ECT the highest magnitude is in the white matter of both temporal lobes and white matter connecting the frontal lobes including the external and internal capsules as well as the anterior corona radiata.

### 3.3 Differences in electric fields between RUL and BL ECT

**Fig. 3:**
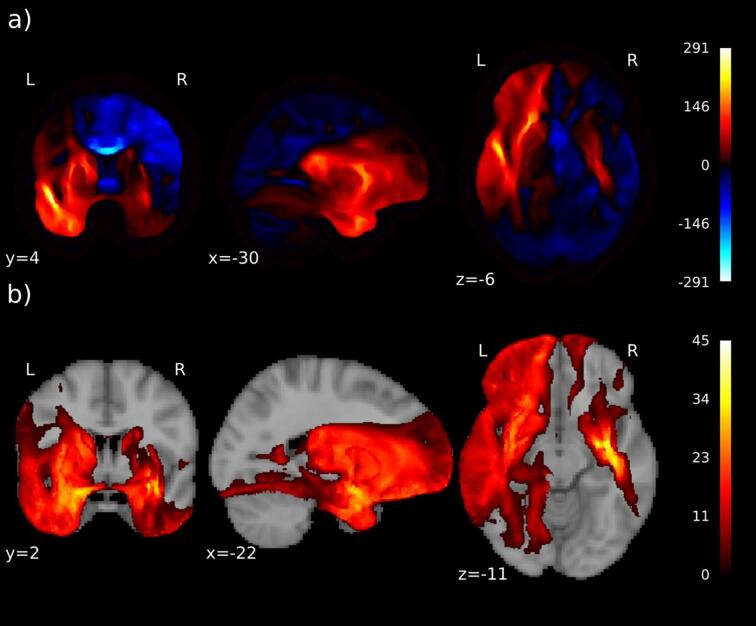
Effect of different electrode placement on electric field. In particular, in the left hemisphere, but also in part of the temporal lobe of the right hemisphere, the electric field for bilateral (BL) electrode placement is significantly larger than in right unilateral (RUL) placement: a) Mean difference of the magnitude (V/m) of electric field for BL vs RUL stimulation in the group who switched stimulation; positive is where BL is larger than RUL (red color); b) T-values for the statistically significant voxels (p < 0.05). (For interpretation of the references to color in this figure legend, the reader is referred to the web version of this article.)

We compared simulated electric fields between RUL and BL electrode placement in patients that received both electrode placements. The mean difference of the electric field due to different electrode placements (BL versus RUL) is shown in Fig. 3a, and statistically significant differences are shown in Fig. 3b. The magnitude of the electric field for BL-treated patients is significantly larger (p < 0.05) in the left hemisphere as well as in part of the temporal lobe of the right hemisphere.

### 3.4 Electric field distribution related to ECT-outcome

**Fig. 4:**
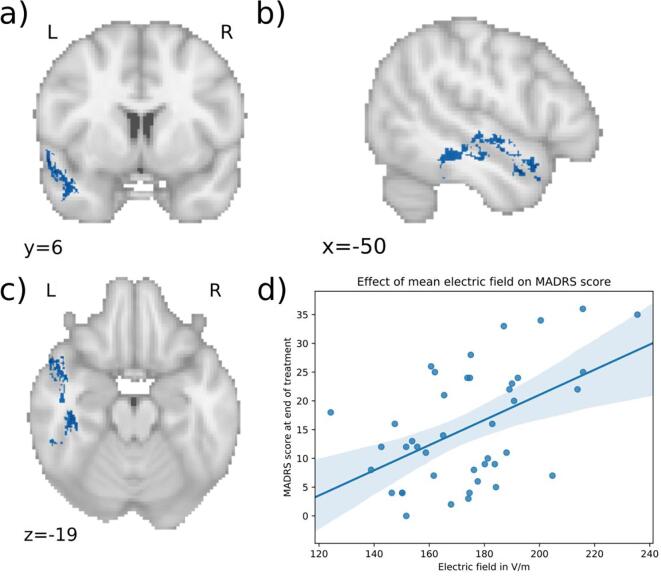
Significant cluster 1 associated with clinical outcome. a), b) c) Higher electric field in the blue areas is associated with less optimal outcome in patients treated with bilateral (BL) ECT (MNI coordinates of peak: −57, 7, −11). The cluster is in the left temporal lobe including parts of the inferior and superior longitudinal fasciculus, inferior temporal gyrus and superior temporal gyrus. d) Scatter plot showing the mean electric field strength of the significant cluster and the end-MADRS-scores, corrected for covariates (for visualization purposes). The higher the electric field in this cluster, the higher was the MADRS-score after treatment. (For interpretation of the references to color in this figure legend, the reader is referred to the web version of this article.)

**Fig. 5:**
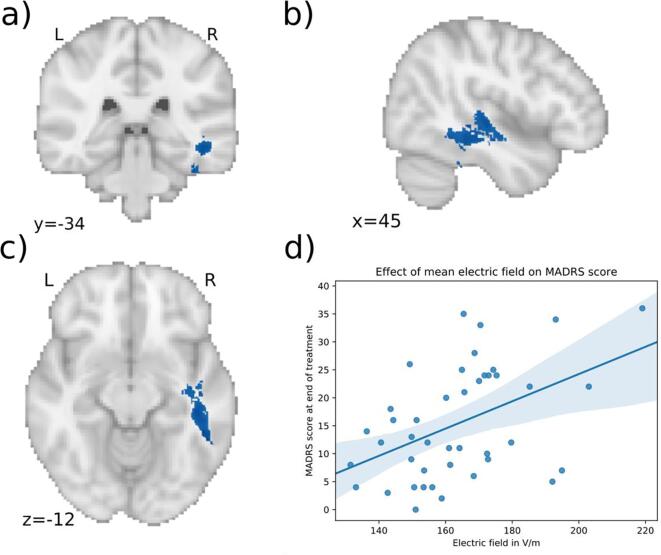
Significant cluster 2 associated with clinical outcome. a), b) c) Higher electric field in the blue areas is associated with less optimal outcome in patients treated with bilateral (BL) ECT (MNI coordinates of peak: 41, −26, –23). The cluster is in the right temporal lobe including parts of the inferior longitudinal fasciculus, superior temporal gyrus and fusiform cortex. d) Scatter plot showing the mean electric field strength of the significant cluster and the end-MADRS-score, corrected for covariates (for visualization purposes). The higher the electric field in this cluster, the higher was the MADRS-score after treatment. (For interpretation of the references to color in this figure legend, the reader is referred to the web version of this article.)

**Fig. 6:**
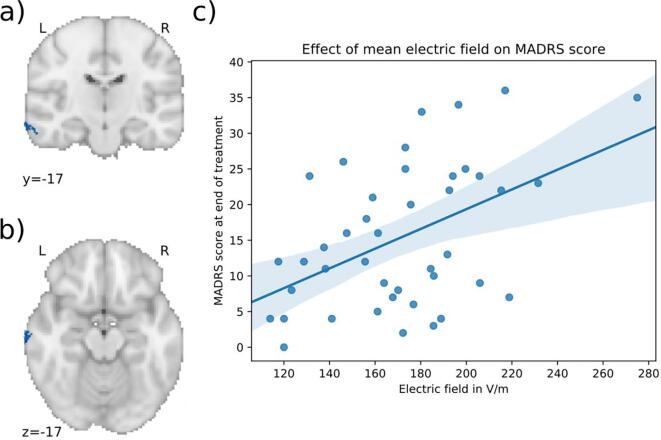
Significant cluster 3 associated with clinical outcome. a), b) c) Higher electric field in the blue areas is associated with less optimal outcome in patients treated with bilateral (BL) ECT (MNI coordinates of peak: −71, −17, 16). The cluster is in the left middle temporal gyrus. d) Scatter plot showing the mean electric field strength of the significant cluster and the end-MADRS-score, corrected for covariates (for visualization purposes). The higher the electric field in this cluster, the higher was the MADRS-score after treatment. (For interpretation of the references to color in this figure legend, the reader is referred to the web version of this article.)

In RUL treated patients, there were no significant differences of electric field distribution between the responders versus the non-responders. In BL patients, there was a significant positive association between the electric field strength and the end-MADRS-score, while correcting for age, sex, baseline MADRS-score, total number of ECT-sessions, mean seizure threshold and whether patients switched electrode configurations during the ECT-course. The statistically significant electric field clusters are presented in Table 2 and are illustrated in Fig. 4, Fig. 5, Fig. 6. Average effect sizes using semi-partial correlations for each cluster and each predictor are listed in appendix Table 1. The first cluster (Fig. 4, p = 0.0422) was in the left temporal lobe. It mostly followed temporal white matter along the left inferior longitudinal fasciculus. It also included parts of the superior longitudinal fasciculus along with the inferior and superior temporal gyri. Parts of it extend into the temporal fusiform cortex and temporal pole. The peak of the cluster (MNI: −57, 7, −11) was in the left temporal pole. The average effect size for this cluster was a R^2^ of 0.53 with the semi-partial R^2^ of the electric field at 0.3 (appendix Table 1). The second cluster (Fig. 5, p = 0.0432) was situated in the right temporal lobe and followed mostly the right inferior longitudinal fasciculus with parts extending into the temporal fusiform cortex and superior temporal gyrus. The peak (MNI: 41, −26, –23) was in the right temporal fusiform cortex. This cluster had an average R^2^ of 0.53 with the electric field semi-partial R^2^ of 0.29. The third cluster (Fig. 5, p = 0.0468) was in the left middle temporal gyrus (MNI: −71, −17, −16) with an R^2^ of 0.55 and for the electric field a semi-partial R^2^ of 0.32. All of the clusters were positively associated with the MADRS-score after the ECT-course, indicating that higher electric field in the clusters was associated with less optimal treatment outcome. This can be seen in Fig. 4d, 5d and 6d where for visualization purposes the mean electric field in the clusters is plotted against the MADRS-score after treatment, with covariates regressed out. In these clusters, the range of the electric field strengths was 120 – 275 V/m.

**Table 2:** Significant electric field clusters. Statistically significant electric field clusters predicting Montgomery-Åsberg Depression Rating Scale (MADRS) score after the ECT-course. Higher electric field was associated with higher MADRS-scores, suggesting less optimal ECT-outcome.

**Cluster no**		**Size mm^3^**	**MNI coordinates peak**	**P-value of peak**	**Regions with majority of voxels**	**Region of peak**
1		6034	(−57, 7, −11)	0.0422	Left inferior and superior longitudinal fasciculus, superior temporal gyrus, inferior temporal gyrus, fusiform cortex, temporal pole	Temporal pole
2		4566	(Deng et al., 2011, van et al., 2013, Sackeim, 2004)	0.0432	Right inferior longitudinal fasciculus, superior temporal gyrus, fusiform cortex	Right fusiform cortex
3		464	(-71, −17, −16)	0.0468	Left middle temporal gyrus	Left middle temporal gyrus

## 4 Discussion

In this study, the relation between computer-modeled distributions of the electric field strength to clinical outcome was explored in 67 ECT-patients. The electric field distribution varied remarkably between patients and especially between the different electrode placements that were used. In RUL ECT, the highest electric fields were seen in the right hemisphere and in the corpus callosum; in BL ECT, the highest electric fields were in the white matter of both temporal lobes and a white matter connection of the frontal lobes. Comparing patients treated with RUL and BL electrode placement, electric fields in BL ECT were shown to be significantly higher than in RUL in the left hemisphere as well as in part of the temporal lobe of the right hemisphere. In RUL ECT, spatial electric field strength did not differ between responders and non-responders. However, in BL treated patients, higher electric field strength in the temporal lobes appeared to be associated with less optimal ECT-outcome. These results support the long-standing hypothesis that the response to ECT may be related to individual differences in brain anatomy that determine the distribution of the electric field (Sackeim, 2004).

### 4.1 Distribution of electric fields in ECT-patients

Little is known about the distribution of electric fields within the human brain during ECT. Understandably, it is impractical to measure actual electric fields in brains of living ECT-patients. Currently, the use of MRI in patients to construct computer-models is, therefore, the best there is to explore electric fields during treatment of actual patients. Individuals in our study showed large variations in modeled brain electric field distributions, more prominent depending on the used electrode placement (see Fig. 1). In RUL electrode placement, highest electric fields were shown in the right hemisphere, but also deeper within the corpus callosum as main connection between both hemispheres (see Fig. 2a); in BL electrode placement highest electric fields appeared in both temporal lobes, but also in the white matter connections within the frontal lobes (capsula interna/externa and corona radiate; see Fig. 2c). Logically, the modeled electric fields appeared to be higher directly beneath the applied electrodes, but higher electric fields in the deeper laying connecting structures appear less obvious. Moreover, in BL ECT, the modeled electric field strengths were significantly higher than in RUL placement, especially in the left hemisphere but also in parts of the right temporal lobe (see Fig. 3). The clinical consequences of electrical stimulation of certain brain areas remains unknown. But for decades it is suggested that electrical stimulation of the deeper parts of the brain is associated with better ECT-efficacy and stimulation of the temporal lobes with cognitive side-effects (Sackeim et al., 1993).

### 4.2 Association between spatial electric field strengths and ECT-outcome

For BL electrode placement, but not RUL electrode placement, the strength of the spatial electric field in the temporal lobes, mostly in white matter, predicted clinical outcome. The effect sizes in the clusters were a R^2^ of around 0.5 with a semi-partial R^2^ of the electric field of 0.3, indicating that in the clusters the electric field explains most of the variance of the model. Note however the effect sizes are calculated after a multiple comparison procedure selecting those voxels where the electric field is associated with the outcome. So the effects will be optimistically biased towards the criteria of that selection (Kriegeskorte et al., 2009). The RUL result is in line with the study of Argyelan et al. (Argyelan et al., 2019) which did not find an association between RUL electric fields and treatment outcome. They, however, only looked at this relationship in gray matter regions of interest while we use a voxel wise whole brain approach, not excluding white matter. The role of white matter in inducing, propagation and termination of seizure activity, more specific in ECT, is still not clear. A variable influence of white matter in individual patients, though, seems imaginable. Interestingly, our study showed associations of ECT-outcome and electric field strengths in both sides of the inferior longitudinal fasciculus (ILF) and the left middle temporal gyrus (LMTG). The ILF connects the occipital- and the anterior temporal-lobes of the human brain and was functionally associated with object- and face-recognition, alexia and semantic impairment (Herbet et al., 2018). The middle temporal gyrus was associated with semantic memory and language processing (Xu et al., 2015). In ECT, patients may show short-term temporally aphasia directly after the seizure and sometimes may suffer more long-term executive dysfunctions due to retrograde amnesia (e.g., temporally forgetting well-known faces or the route to the supermarket). In daily clinical practice, though, (much) improvement of mood with ECT may be present despite of (severe) cognitive side-effects. Therefore, it is speculative whether cognitive side-effects or less ECT-effectiveness are caused by direct electrical stimulation of these specific brain areas.

In clinical practice, once RUL ECT appears not to be successful, switching to BL ECT is often beneficial although often patients experience more cognitive side-effects. In case of switching, the clinician will stimulate the patient bilaterally with lower charges than when continuing with RUL ECT. Regardless of the reduction of the amount of charge, our results show that the brain still receives stronger electric fields with BL stimulation. But in addition, higher electric fields in specific brain areas are associated with higher MADRS-scores after a course of BL-ECT (see Fig. 4, Fig. 5, Fig. 6). This paradoxical result suggests that there may be an inverted U-shape relation between electric field dose within the temporal lobe white matter and clinical response: the administration of a weaker electric field during RUL-ECT does not provide optimal clinical benefit and will require switching to BL electrode placement, but in BL-ECT a too strong electric field in the temporal lobes may deliver less benefit compared with moderately strong electric fields. In contrast to the hypothesis that stimulation of deeper midline and frontal regions affects efficacy, our results indicate that the temporal lobes may be important for this relationship. Whether this relates to more cognitive side-effects remains unclear, because we did not have appropriate measures of cognition in our study; this is also a general limitation in present-day ECT research. Additionally, more (or more severe) cognitive side-effects may have resulted in earlier discontinuation of the ECT-course accompanying higher MADRS scores after treatment. Therefore, discovering the relationship between spatial electric field strengths, effectiveness and cognitive side-effects of ECT will be very helpful in the shared decision-making process to initiate ECT.

We did not observe any negative correlations between the electric field and treatment outcome. A negative correlation would have indicated regions where higher electric field would result in better outcome. As to why this was not observed there could be multiple reasons. Firstly, our study could have lacked power to detect the negative correlations. Furthermore, ECT has through the years already been optimized by modifying various stimulus parameters. So, a ceiling to how effective the treatment can be may be present. Thirdly, the current amplitudes in use (800 – 900 mA) have been shown in models to exceed the neuronal activation threshold of the entire brain by more than sixfold (Deng et al., 2011). Therefore, it could be that there is not much gained by increasing the stimulus amplitude (and thus the electric field) further. There have also been small studies indicating that low amplitude seizure therapy using 500 mA might be effective (Youssef and Sidhom, 2017, Youssef et al., 2019) A recent trial comparing various stimulus amplitudes (600 – 800 mA) did, however, find that 600 mA resulted in worse ECT-outcome relative to 700 and 800 mA (Abbott et al., 2020). More studies are definitely needed in this area.

### 4.3 Strengths and weaknesses

Our study in 67 ECT-patients is associated with certain strengths and limitations. We developed a new processing pipeline to enable the current analysis, in which we use the neuroimaging data as independent variable instead of dependent variable. In addition, the sample size was relatively large and consisted of a typical ECT-population, and is therefore representative for the clinical population. On the other hand, this is an association study and as such there are various clinical confounders and MRI technical limitations that need to be addressed. Because this is an observational study, inferences about the causal nature of the effect of electric field on ECT-outcome is difficult.

#### 4.3.1 Clinical confounders

The included ECT-population was highly heterogeneous, and patients had various symptoms in addition to the MDE, such as psychotic features, somatic comorbidities, and suicidality, which may have affected the ECT-outcome. Moreover, variables such as age and sex are known to influence treatment outcome and may have affected the patients’ modeled electric fields through effects on head size and anatomy. We could include these in our statistical model, as well as the baseline severity of the MDE which may have impacted the ECT-outcome. There may have been outcome effects of the concomitant medication use of the patients during the ECT-course, which we were not able to correct for. Unfortunately, no appropriate baseline and outcome measurements of cognitive functioning were obtained in our patients. These missing data precluded the analyses of cognitive side-effects in relation to spatial electric field distributions in our study.

#### 4.3.2 MRI technical limitations

Using only T1-weighted images very likely decreased the anatomical accuracy of the head models and by that caused spurious increases of interindividual differences of the electric fields (Puonti et al., 2020). Our data was acquired using a 1.5 T scanner; the noise quality of the images is suboptimal for head model construction, which generally recommends the use of 3 T images (Nielsen et al., 2018). The inclusion of T2-weighted images would have improved tissue segmentation, particularly skull segmentation. Given the limitation of only having T1-weighted images, we are using the ‘headreco’ command in SimNIBS, which uses the SPM12 with the CAT12 toolbox to conduct tissue segmentation, which has been shown to outperform the Freesurfer or SPM12-only segmentation routines (Nielsen et al., 2018). The CAT12 toolbox also computes an image quality rating (IQR) based on various measures including image resolution, noise contrast ratio, and inhomogeneity contract ratio. The average IQR for our dataset is 71.7% ± 4.8%, which is qualitatively satisfactory for the purpose of tissue segmentation (http://www.neuro.uni-jena.de/cat12-html/cat_methods_QA.html#Dahnke:2016).

The registration to MNI space will not be perfect, so that – for example - gray matter from one subject will overlap with white matter of another subject. These registration errors artificially increase the interindividual variability of the electric field in many parts of the gray matter and also in white matter close to their boundary, which will weaken any relationship between the electric field with clinical variables of interest for these positions. The effect may occur less in deeper white matter regions. This is also observed as higher standard deviations in the gray matter in Fig. 2b and 2d. Also, the anisotropy of electrical conduction along white matter fibers is known to affect the electric fields strength (Lee et al., 2012). As quantified in Lee et al. (Lee et al., 2012), comparing the electric field strength in a head model with isotropic versus anisotropic white matter conductivity, the relative error over the whole brain is 18% for BL ECT and 7% for RUL ECT. Unfortunately, our dataset did not include individual diffusion MRI data, so we could not account for this effect. In addition to using isotropic conductivity values, we also used a fixed set of tissue conductivity values. While the use of ‘standard’ tissue conductivity values generally results in simulated electric field values with good correspondence to *in vivo* measurements (Opitz et al., 2016, Huang et al., 2017), there are expected uncertainties and interindividual variation in tissue conductivity that can affect the electric field strength calculations. A recent method was developed using generalized polynomial chaos expansion to quantify the effect of conductivity variability on the induced electric field (Saturnino et al., 2019). A sensitivity analysis should be included in future studies.

Although we used a sophisticated computer model to estimate individual electric fields in the brain, the real electric fields could not be measured and will therefore remain unknown. The electrode placements were standardized by experienced ECT-psychiatrists, but there would have been slightly differences between patients and between treatment sessions that the models could not account for. Unfortunately, we were not able to mark the exact position of the applied electrodes for each patient in the included MRI-scans, which may have influenced our results. In a previous simulation study on the importance of precise electrode placement for targeted TES (Opitz et al., 2018) the authors investigated the effect of electrode movement in 25 head models of healthy individuals, carried out using very similar methods to our study, with comparable temporal region electrode placement. Their results suggest that with 1-cm-diameter electrodes, 1 cm deviation of electrode movement in the inferior–superior or anterior–posterior directions leads to an approximately 10% decrease in the electric field distribution correlation value. Within a 1 cm deviation, electric field distributions are highly similar. In addition, the effect of inter-electrode distance on the maximum induced electric field is dependent on electrode diameter (Deng et al., 2013). In general, the maximum electric field is less sensitive to changes in electrode spacing with larger diameter electrodes. Specifically, with two 5-cm diameter ECT disc electrodes, spaced more than 5 cm apart (as is the case with our RUL and BL placements), the effect on maximum electric field due to variation in inter-electrode spacing is generally small (Deng et al., 2013).

### 4.4 Future directions

An inverted U-shape relation between electric field in right temporal white matter and clinical outcome may enable the development of patient-specific ECT protocols. For example, future electrode placements may spare these areas more thoughtfully, which may lead to a further optimization of ECT. Also, clinicians may select the preferred electrode placement in advance, by choosing a placement which preserves the temporal lobes most effectively. For example, an ongoing first-in-human study is now testing the safety and feasibility of focal ECT using a multielectrode array (https://clinicaltrials.gov/ct2/show/NCT03895658). This, coupled with targeting algorithms (i.e., (Dmochowski et al., 2011), may yield optimal electrode configurations that can deliver electric field selectively to target brain regions in individual patients. Also, it may be useful to examine treatment optimization approaches, such as used in other TES strategies (i.c., tDCS (Saturnino et al., 2020), in ECT studies as well. Finally, ECT modeling studies generally assume the magnitude of the electric field to be the key dosing metric (see justifications in (Deng et al., 2011), the directionality of the electric field vector could also be an important determinant of outcome. At the mechanistic level, an investigation of the electric field directionality effects would call for development of direction-sensitive neural activation models. From a biomarker discovery standpoint, future work could consider the use of machine learning approaches to detect local electric field features relevant to clinical outcome.

## 5 Conclusion

Using a computer model derived from individual MRI data, electric field distributions in brains of ECT-patients showed significant variability between patients, especially comparing RUL and BL electrode placement. Electric field strength was higher in BL than in RUL electrode placement, particularly in the left hemisphere and part of the right temporal lobe. Less optimal ECT-outcome was associated with higher electric field strength in the temporal lobes, particularly in white matter. This indicates an inverted U-shape relation and implies that overstimulation of the temporal lobes may hamper a good clinical outcome. If replicated, individualized pre-ECT computer-modelled electric field distribution may enable the development of patient-specific ECT protocols using multi electrode arrays to control better which regions are stimulated.

## Funding and disclosure

The authors have no conflicts of interest to report regarding the subject of this study.

## CRediT authorship contribution statement

**Egill Axfjord Fridgeirsson:** Conceptualization, Methodology, Software, Writing - original draft, Writing - review & editing. **Zhi-De Deng:** Conceptualization, Methodology, Software, Writing - review & editing. **Damiaan Denys:** Supervision, Writing - review & editing. **Jeroen A. van Waarde:** Conceptualization, Supervision, Writing - review & editing. **Guido A. van Wingen:** Conceptualization, Supervision, Writing - review & editing.

## Declaration of Competing Interest

The authors declare that they have no known competing financial interests or personal relationships that could have appeared to influence the work reported in this paper.
