# Supplementary material for: Electric field strength induced by electroconvulsive therapy is associated with clinical outcome
Source: Neuroimage Clin. 2021 Feb 9;30:102581. doi: 10.1016/j.nicl.2021.102581 (PMC7895836; doi:10.1016/j.nicl.2021.102581)
Supplement: Supplementary data 1 [file mmc1.docx]

**Supplementary Table 1: Average effect sizes of each cluster in form of R^2^ and semi-partial R^2^**

| **Cluster no** | **Average explained variance of model (R^2^)** | **Average explained variance of each predictor (Semi-partial R^2^)** | | | | | | | | | | | | |
| --- | --- | --- | --- | --- | --- | --- | --- | --- | --- | --- | --- | --- | --- | --- |
|  |  | **E-field** | | **Age** | | **Sex** | **Baseline MADRS** | | **Sessions** | | **Mean seizure threshold** | | **Switcher** | |
| 1 | 0.53 | 0.3 | | 0.02 | | 0.06 | 0.01 | | 0.05 | | 0.06 | | 0.12 | |
| 2 | 0.53 | 0.29 | | 0.001 | | 0.007 | 0.008 | | 0.005 | | 0.04 | | 0.22 | |
| 3 | 0.55 | 0.32 | 0.001 | | 0.0 | | | 0.014 | | 0.007 | | 0.04 | 0.13 |  |


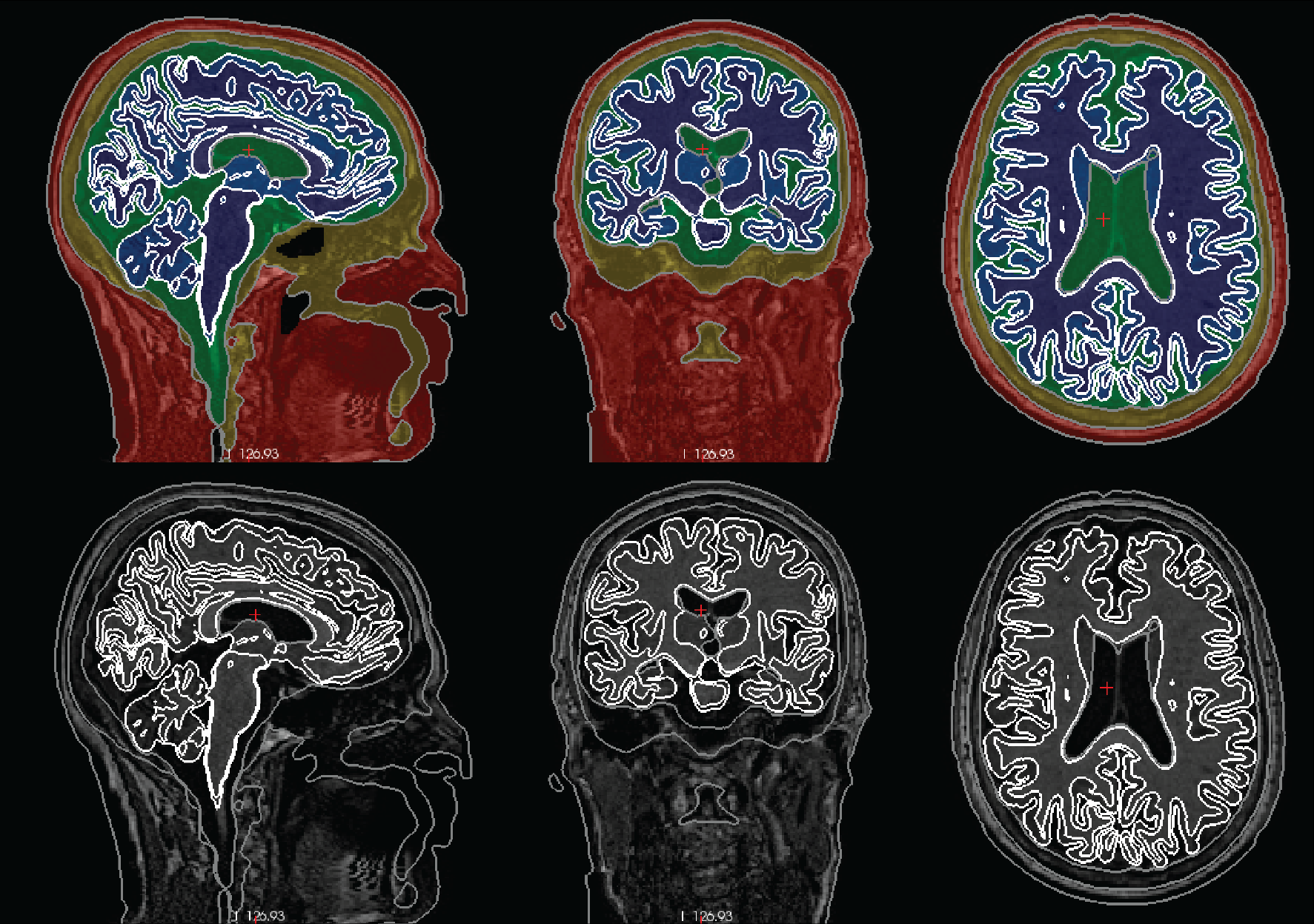


**Supplementary Figure 1 – A representative example of the head model segmentation.**
